# P92 - The prevalence of food sensitisation in children suffering from eczema

**DOI:** 10.1186/2045-7022-4-S1-P147

**Published:** 2014-02-28

**Authors:** Agathi Karagiannidou, Ioannis Xinias, Sofia Botskariova, Nikolaos Karantaglis, Olympia Ourailoglou, Maria Fotoulaki, Maria Emboriadou, Aristidis Deligiannidis, Antigoni Mavroudi

**Affiliations:** 1 3rd Pediatric Department, Aristotle University, Hippocration Hospital, Thessaloniki, Greece; 2 4th Pediatric Department, Aristotle University, Papageorgiou Hospital, Thessaloniki, Greece

## Background

Atopic dermatitis (eczema) is a highly pruritic chronic inflammatory skin disease. Food allergy has been strongly correlated with the development of persistence of atopic dermatitis.

## Aim of the study

To investigate the association of food allergy in Greek children with atopic dermatitis.

## Patients and methods

Eighty-eight (88) children with eczema (59 boys and 29 girls) aged between 12 months and 6 years were studied. All the children underwent allergological investigation with assignment of specific IgE antibodies (Elisa Method) to the following food allergens: α- lactalbumin, β-lactoglobulin, casein, milk proteins, egg white, egg yolk, beef, soy, wheat, and cod.

## Results

Food sensitization occurred in 39 out of 88 children (44,3%). The frequency distributions for elevated specific IgE antibodies to various food allergens in children with eczema are shown in the following Table (Table 1).

**Table 1:** Frequency distributions of food sensitization in children with eczema

Milk proteins	27	25,00%
Egg white	22	21,00%
a-lactabumin	21	20,00%
Egg yolk	12	11,00%
β-lactoglobulin	10	9,00%
Wheat	8	7,00%
Casein	3	3,00%
Soy	3	3,00%
Cod fish	1	1,00%
Beef	0	0%
**Total**	**39**	**100%**

## Conclusions

Food sensitization has a high prevalence of almost 44% among children with eczema.

Milk proteins are the most common food allergens implicated in children with eczema (27.25%), followed by egg white (22.21%), a-lactalbumin (21.2%), egg yolk (12.11%), β-lactalbumin (10.9%) and wheat (8.7%).

Beef, soy and cod fish are less common food allergens in children with eczema.

